# Targeting maternal gut microbiome to improve mental health outcomes—a pilot feasibility trial

**DOI:** 10.3389/fpsyt.2024.1414291

**Published:** 2024-08-07

**Authors:** Faith Gallant, Kieran Cooley, Sophie Grigoriadis, Neda Ebrahimi

**Affiliations:** ^1^ Rehabilitation Nevena Christina Case Management and Occupational Therapy Services (NCCO), Toronto, ON, Canada; ^2^ Department of Research and Clinical Epidemiology, Canadian College of Naturopathic Medicine (CCNM), Toronto, ON, Canada; ^3^ Department of Human Biology, University of Toronto, Toronto, ON, Canada; ^4^ National Center for Naturopathic Medicine, Southern Cross University, Lismore, NSW, Australia; ^5^ Women’s Mood and Anxiety Center, Reproductive Transitions, Department of Psychiatry, Sunnybrook Health Sciences, Toronto, ON, Canada; ^6^ Department of Psychiatry, University of Toronto, Toronto, ON, Canada

**Keywords:** perinatal mental health, gut microbiome, nutritional counseling, omega-3 fatty acids, probiotics, dietary fiber, perinatal depression, perinatal anxiety

## Abstract

**Background:**

Perinatal depression and anxiety (PDA) is prevalent in new and expectant mothers, affecting millions of women worldwide. Those with a history of mood and anxiety disorders are at the greatest risk of experiencing PDA in a subsequent pregnancy. Current safety concerns with pharmacological treatments have led to a greater need for adjunctive treatment options for PDA. Changes in the composition of the microbiome have been associated with various diseases during pregnancy, and these changes are thought to play some role in perinatal mood disorders. While the relationship between PDA and the microbiome has not been explored, evidence suggests that nutritional interventions with fiber, fish oils, and probiotics may play a favorable role in neuropsychiatric outcomes during and after pregnancy. The primary objective of the present study is to assess the feasibility and acceptability of a combination of nonpharmacological interventions to maintain stability in pregnant women who have a history of depression and/or anxiety. This study will also aim to understand ease of recruitment and protocol adherence in this cohort.

**Methods:**

This is a single-centered, partially randomized, placebo-controlled, double-blind feasibility trial. One hundred pregnant women with a history of depression and/or anxiety/PDA will be recruited and randomized into one of four arms, which could include the following: receiving a daily dose of both investigational products and dietary counseling on increasing dietary fiber, receiving a daily dose of both investigational drugs only, receiving fish oil investigational product and placebo, and a control arm with no intervention. The study involves six study visits, all of which can be conducted virtually every 3 months from the time of enrollment. At all study visits, information on diet, mental health, physical activity, and sleep quality will be collected. Additionally, all participants will provide a stool sample at each visit.

**Discussion:**

It is anticipated that pregnant women with a history of depression and anxiety will be particularly interested in partaking in this trial, resulting in favorable recruitment rates. Given the positive findings of omega-3 fatty acids (O3FAs) and probiotic supplements on mental health symptoms in nonpregnant adults, we expect a similar trend in PDA symptoms, with a low likelihood of adverse events. This study will build the foundation for larger powered studies to further contribute evidence for the efficacy of this potential preventative treatment option.

**Trial registration:**

This trial was registered at ClinicalTrials/gov on October 6, 2023; NCT06074250. Trial Sponsor: The Canadian College of Naturopathic Medicine, 1255 Sheppard Ave E, Toronto, ON M2K 1E2, 416-498-1255.

## Background

Perinatal depression and anxiety (PDA) is prevalent in new and expectant mothers, affecting millions of women worldwide. Recent evidence suggests that one in seven women in the 20th–28th week of gestation develop depression, which impacts their ability to return to normal functioning; in the same period of gestation, anxiety has a prevalence of about 8.5%–10.5% (1). A recent meta-analysis included nine studies, with a total of 4,449 participants, examining prevalence rates of generalized anxiety disorder throughout pregnancy. Studies took place in Germany, Brazil, Italy, the UK, Turkey, Nigeria, France, and Sweden. Prevalence rates were found to range from 0.9% to 22.7%, with pooled data analysis revealing an overall prevalence of 3% (2). The pooled prevalence of antenatal depression reported in a 2021 meta-analysis of 173 studies published before 2019 was 20.7% (3). Well-known risk factors for perinatal depression include poor social support, chronic and persistent health issues in their infant, an abusive partner, marital difficulties, familial history, a history of violence, and other negative life events (4). The strongest predictor of perinatal depression, however, is a history of mood or anxiety disorders (4). Those with a history of depression have a 20-fold higher risk of a subsequent pregnancy (5), and relapse rates of 40% have been observed (4). Risk factors for new-onset perinatal anxiety can include low education, living with extended family members, a family history of psychiatric disorders, a history of sleep disorders, multiparity, and other negative life events. However, the most significant risk factor for worsening perinatal anxiety was having a comorbid psychiatric disorder, such as depression (6). Available treatment options for these disorders in the peripartum population include pharmacotherapy and psychotherapy. However, safety concerns for pharmacological treatments during pregnancy and lactation, limited access to care for psychotherapy, and the stigma of mental illness are major barriers to implementing these treatment options (7). Moreover, lack of treatment is associated with morbidity for the mother, infant, and family system and impairment in quality of life (7). Acceptable and readily available treatment alternatives are urgently required.

There is emerging evidence that the gut–brain axis (GBA) is a reasonable target for the management of PDA disorders. The GBA is a recently realized phenomenon in medicine, suggesting continuous bidirectional communication between the gut microbial community and the brain, a requirement for achieving and maintaining gastrointestinal homeostasis and cognitive function. Dysbiosis—an imbalance in bacterial composition—has been implicated in many disorders, including mental health (8). Thus, optimizing the health of the gut microbiome (i.e., treating dysbiosis) may be a logical approach to preventing and treating mood disorders.

Pregnancy itself has been associated with changes in the gut microbiome driven by immunological, hormonal, and metabolic changes—these changes are proinflammatory in nature, leading to an increase in cytokines and leukocytes and the recruitment of other immune cells (macrophages, natural killer cells, etc.) that aid in angiogenesis, transport of respiratory gases and nutrients between mother and fetus, and protection against pathogens (9). Interestingly, certain changes in the composition of the microbiome have been associated with various diseases during pregnancy, such as gestational diabetes, preeclampsia, fetal growth restriction, and obesity (9). Likewise, these changes are thought to be at least partially at play in perinatal mood disorders. A 2022 mini-review examined the correlation between perinatal depression and dysbiosis of the mother’s microbiome and reported dysbiosis as a likely precipitating factor in the development of psychiatric disorders during pregnancy (10). Emerging evidence suggests that nutritional interventions, with fish oils, probiotics, and prebiotics may play a favorable role in neuropsychiatric outcomes (11–16) and that some of these effects are mediated at least partially, by restoring eubiosis.

While not yet examined in a pregnant population, the protective effects of specific nutrients and diets have been demonstrated in major depressive disorder (MDD) in nonpregnant adults. For example, the onset of depression has been shown to increase with high-fat Western diets and significantly decrease with Mediterranean diets (8). The latter has demonstrated a reduction in oxidative stress and an increase in neurotransmitters serotonin, noradrenalin, dopamine, and monoamines, all of which play a role in major depression (8). The higher intake of plant-based fiber and omega-3 fatty acids (O3FAs) in Mediterranean diets may partly be responsible for this protective effect. Dietary fiber is fermented by cecum and large-intestine bacteria. In addition to changing the abundance of specific strains of bacteria, it also drives the levels of short-chain fatty acid production (17). Several observational studies have linked fiber intake to a reduction in the severity of depression as well as depression onset. Mechanisms proposed for this outcome include increases in levels of certain neurotransmitters, neurotrophic factors (e.g., brain-derived neurotrophic factor), and reductions in inflammatory biomarkers (17).

Specifically in the pregnant population, a recent retrospective cohort analysis examined the results of three studies (a randomized controlled trial of a low-glycemic-index diet in pregnancy, the pregnancy exercises and nutrition research study, and a randomized controlled trial on probiotics). Together, data from 1,521 participants were included in this analysis, which showed that the average daily intake of fiber was statistically significant with regard to maternal well-being (*r* = 0.13; *p* < 0.01). Results indicated that nutrients in whole grains, fruits, and vegetables were associated with improved mental health, and the authors suggested that the high fiber content in such foods is the source of these observed benefits (18).

Multiple randomized controlled trials (RCTs) have investigated probiotics in pregnancy. In 2017, Slykerman et al. reported significantly lower postpartum depression and anxiety scores in women taking probiotics versus placebo (19). However, this analysis was done retrospectively as a secondary outcome. A 2020 RCT by Dawe et al. found no impact on psychiatric outcomes of obese women at 26 weeks of gestation. However, this sample had lower baseline mental illness scores than what would have been expected, and there was no postpartum assessment (20). In 2021, Browne et al. completed a pilot RCT and concluded that the impact of probiotics on prenatal maternal anxiety and depression is feasible and acceptable. While depression and anxiety symptoms decreased after the intervention, the authors found no significant difference compared to placebo and concluded that the small sample size (*n* = 40) may have underpowered the design (21).

Recent evidence points to O3FAs having an impact on the composition of the microbiome. In rodent models, maternally separated rats with increased stress markers have an altered composition of bacteria in their microbiome, including decreased numbers of bacteria in the *Lactobacillus* genus and elevated numbers of *Oscillibacter*, *Anaerotruncus*, and *Peptococcus* general (22). Pusceddu et al. showed that with long-term administration of eicosapentaenoic acid (EPA)/docosahexaenoic acid (DHA) in stressed rodents, there is a restoration of the microbiome composition and reduction of inflammatory processes typically associated with stress (23). Specifically, EPA/DHA supplementation appeared to restore the microbiome composition to a state similar to that of nonstressed rodents, including increased *Lactobacillus* genus and reduced *Anaerotruncus* genus (23).

Currently, data linking O3FA administration with benefits in mood disorders through microbiota modulation is mostly limited to rodent models. However, the positive findings from observational and clinical trials on the impact of O3FAs on mood disorders call for a closer look at the interaction of fish oil with the microbiome and its impact on the GBA of pregnant women (24).

### Rationale for study

Evidence for the positive role of fiber, probiotics, and O3FAs in depression and anxiety is accumulating. Studies on the combination of these supplements and their mitigating effects on the microbiome remain elusive. A significant knowledge gap remains on the usability and efficacy of fiber, probiotics, and O3FAs in the pregnant population, particularly in relation to mental health disorders. Moreover, challenges exist in the design and conduct of randomized controlled trials in pregnancy or the use of complex, combination interventions, making drawing inferences challenging. Given the nonpharmacological nature of the supplements and their wide availability, safety, and cost-effectiveness, they have a high potential to serve as a preferred management option for the prevention or treatment of mental health disease in pregnant and lactating mothers. Demonstrating the relationship in this population is the first step in using an appropriate research design.

### Objectives

The main objective of this study is to assess the feasibility and acceptability of a combination of nonpharmacological interventions—a high-fiber diet, probiotics, and fish oil supplementation—in currently stable pregnant women with a history of depression and or anxiety. This population has specifically been chosen because a history of mood or anxiety disorders is the strongest predictor of PDA (4).

This study will also aim to understand the ease of recruitment, protocol adherence, and treatment compliance in this cohort. The findings of these objectives will inform on strategies needed to build a larger trial aimed at comparing the efficacy of these interventions and the magnitude of change and impact of the microbiome and PDA.

## Methods

### Trial design and setting

This is a single-centered, partially randomized placebo-controlled double-blind feasibility trial with three intervention arms (Gutopia, Gutboost, and Gutless) and one active control. In total, 100 pregnant women with a history of depression and/or anxiety/PDA attending Sunnybrook Hospital Clinics in the Greater Toronto Area will be recruited.

Participants in Gutopia will receive a daily dose of both investigational products and dietary counseling on increasing dietary fiber. Participants in Gutboost will receive a daily dose of both investigational supplements, while participants in the Gutless arm will receive fish oil and a placebo. Participants in Gutless and Gutboost will be blinded to the status of the placebo/probiotic they will be receiving. The study member interacting with the patients will also be blinded to the treatment group the participant is assigned to. Participants who fully consent to receiving both investigational products, including placebo, will be fully randomized to one of the intervention arms. Participants not willing or able to take a placebo or probiotics will be assigned (not randomized) to the appropriate group that accommodates their preferences/needs. Participants unable or unwilling to take any of our investigational products will be assigned to the active control arm. Women in the control arm will receive standard care offered to all obstetric patients seen at the hospital.

**Figure 1:**
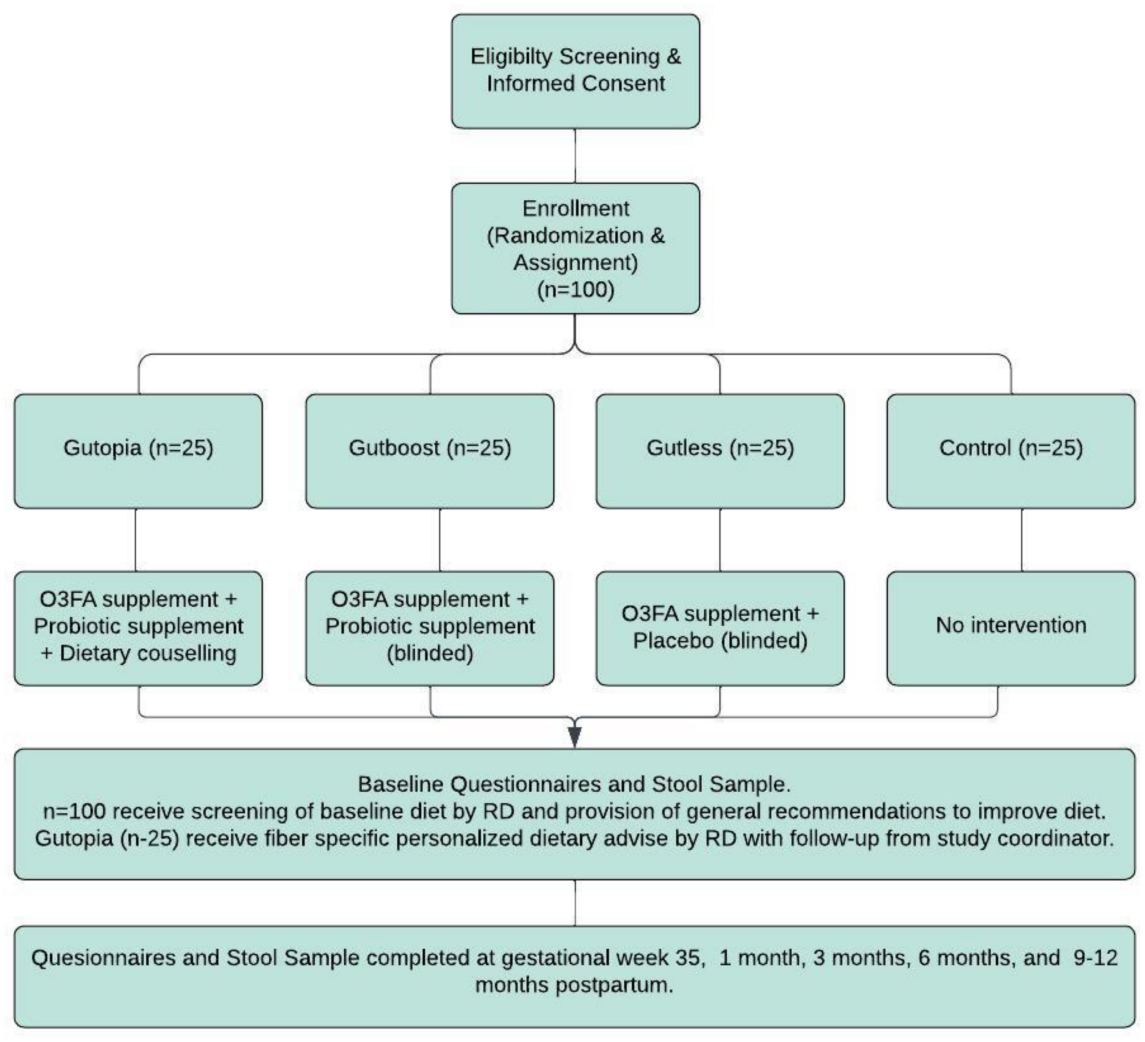
Diagram of participant flow through the trial.

The study involves six study visits, all of which can be conducted virtually every 3 months from the time of enrollment (**Figure 1**). The last study visit will happen between 9 and 12 months postpartum. Two of the study visits will occur during pregnancy [enrollment (visit 1) and third trimester (visit 2)], with the remaining occurring at 1, 3, 6, and 9 months postpartum. At all study visits, information on diet, mental health, physical activity, and sleep quality will be collected. Additionally, all participants will provide a stool sample at each visit, using our stool collection kits, which will be shipped to or dropped off at our center.

The study has been reviewed by the Research Ethics Board of the Canadian College of Naturopathic Medicine and the Research Ethics Board of Sunnybrook Health Sciences Centre. Any amendments to this protocol will be communicated to the Research Ethics Board and Health Canada (the authority responsible for approval, conduct, oversight, and inspection of clinical trials in Canada) and modified on the trial registry.

### Participant eligibility

#### Inclusion criteria

1. A person aged 18–43 years old.
2. At 12–35 weeks of gestation at the time of enrolment.
3. Uniparous pregnancy.
4. A nonsmoker, alcohol, or recreational drug user.
5. Financially stable.
6. In a married or common-law relationship.
7. Clinical diagnosis of lifetime depression/anxiety or PDA (stable since the conception of the current pregnancy).
8. No other significant comorbidities.
9. Ability to read in English and provide informed consent.

#### Exclusion criteria

1. Prepregnancy BMI > 30.
2. Low income (unable to afford basic daily needs).
3. Single parents without any kind of family support.
4. Having a child with significant mental/physical disability.
5. Previously diagnosed with other major mental health disorders.
6. Taking prescription medications (other than SSRIs/SNRIs/TCA).
7. Smoking and recreational drug use.
8. Dietary restrictions or allergies to fish oils.
9. Repeated antibiotic use.
10. Women are unable to switch to study brand supplements.
11. Multiparous women with young children (i.e., one or more children less than 4 years of age at the time of delivery).
12. No/low English reading comprehension.

Our inclusion criteria allow for antidepressant/anxiety use. Medication use is an important prognostic factor that can significantly alter mental health outcomes. Hence, the number of patients treated with an antidepressant/anxiolytic needs to be equally distributed among all arms. To achieve this, we will use stratified randomization using two strata (treated vs. untreated) through a computer-generated process using permuted blocks of three and six. The process will be repeated separately for untreated participants.

Preexisting and diagnosed major mental health disorders will be determined at the time of screening using a modified version of the Mini International Neuropsychiatric Interview (MINI). The MINI, conducted by a trained research team member, will allow for confirmation of a history of anxiety/depression and an absence of current anxiety/depression and other major mental health disorders (bipolar, schizophrenia, psychosis, etc.).

### Sample size, recruitment plan, and prestudy screening

While a consensus on an ‘ideal’ sample size for pilot/feasibility studies has not been reached, our proposed number (*N* = 100) meets the recommendation for best practice for the conduct of pilot trials.

This is a single-center study conducted at Sunnybrook Hospital, in Toronto, Canada. Pregnant patients attending obstetrics clinics or The Women’s Mood and Anxiety Clinic at Sunnybrook Hospital will be informed by their attending healthcare provider about the study, and permission to share their contact number with the research team will be requested. Interested patients will be called by a member of the research team and screened for inclusion/exclusion criteria and their ability/status on O3FA and probiotic intake. Patients not willing or unable to take either supplement will receive information about the control arm of the study. Patients willing to take the study supplements will receive information about the interventional arms of the study and the process of randomization. Participants will also be informed that they may receive a placebo instead of probiotics. This design accommodates patient preferences and expectations and emphasizes the goal of an inclusive, pragmatic study design to generate real-world data.

### Randomization and baseline evaluation

All eligible patients interested in participating will be consented to and randomized or assigned to an intervention or control arm based on responses during screening. If eligible for full randomization, this will be completed by the study principal investigator using a computer-generated randomization via random permuted blocks. The study coordinator will only be informed of the status of randomization to the Gutopia arm and will be blinded to the Gutboost and Gutless arms.

Once randomized, the participant will be scheduled for the baseline assessment as soon as possible. If scheduled as a virtual visit, a stool kit will be mailed to the participant or provided at their prenatal visit. There is no predetermined time between the screening and baseline; however, the baseline visit will take place prior to the 35th gestational week (visit 2). The collection of baseline questionnaires will include the following: The Generalized Anxiety Disorder-7 (GAD-7), The Edinburgh Postnatal Depression Scale (EPDS), Single-Item Sleep Quality Scale, Pregnancy Physical Activity Questionnaire Consumer Financial Protection Bureau—Financial Wellbeing (CFPB-FWB), Demographic Questionnaire, and the Dietary Screening Form. Please see the supplementary materials for a breakdown of the versions used and the psychometric properties of each questionnaire. The baseline visit will also include the provision of a stool sample.

There will be a total of six study visits; visit 1 (baseline) and visit 2 will occur during pregnancy, with the remaining four scheduled to take place in the postpartum period (**Figure 1**). All questionnaires completed at baseline, with the exception of the CFBP-FWB, the Demographic Questionnaire, and the Dietary Screening Form, will be completed at all postpartum visits. A postdelivery questionnaire will be completed at visit 3 (~ 4–6 weeks postpartum), and a postpartum questionnaire will be completed at visits 4–6. The sessions will be 45–60 minutes in length.

### The intervention

Participants in all intervention groups will receive a 200-ml bottle of Genestra brand Super EFA Forte Liquid Omega-3 Fatty Acid. Participants will be instructed to take 1 teaspoon a day with a meal.

Participants in the Gutopia and Gutboost intervention groups will receive HMF Maternity Probiotic Formula, a Genestra brand. Participants will be asked to take one tablet daily with food.

Details of both supplements are provided under Investigational products in the following section.

Only participants in Gutopia will receive a dietary intervention. Intervention is geared toward increasing gut-friendly foods via a flexible dietary plan aimed at increasing daily uptake of prebiotic and probiotic foods. A registered dietitian will use baseline food questionnaire data provided by each subject at enrollment to devise strategies and recommendations for increasing intake of pre/probiotic foods. The target goal is to include at least 35 g/day of fiber and at least one to two servings of fermented foods daily. The dietary intervention will also include recommendations minimizing highly processed foods high in sugar and fat. To improve adherence and accountability, weekly follow-ups by phone, virtual meeting, or email will be conducted by a trained study team member until patients have reached a level of confidence in attaining goals. During each follow-up, the study team will monitor the level of adherence to the study RD’s recommendations. These follow-ups will be designed to provide encouragement while helping patients address and prevent triggers that jeopardize their fiber intake.

#### Use of theory

Given the nature of this feasibility study, we aim to promote increased accountability with regular follow-ups to drive change. To achieve this, theoretical proponents of motivational interviewing will be incorporated into study visits. For instance, behavior change will be enabled by causing participants to verbalize arguments for changes and decrease language that favors the status quo. Furthermore, relational factors, such as expressions of empathy, will be used to promote positive change (25).

#### Educational material and resources

Participants in the Gutopia arm will be presented with food tables listing common foods, and snacks from different food groups, containing the highest amount of fiber per serving. This is to help enable participants to reach their target daily fiber with the fewest servings possible. These tables are categorized by food group and provide the food names, brands, and recommended serving sizes. These have been provided in the supplemental information section.

#### Investigational products

All participants in treatment groups will receive a 200-ml bottle of Genestra brand Super EFA Forte Liquid Omega-3 Fatty Acid. This product contains a total of 2,600 mg of O3FA (1,500 mg of EPA + 1,000 mg of DHA) per 5-ml serving. The participant will be instructed to take 1 teaspoon a day with a meal. Each bottle contains about 40 servings, and a daily consumption of 1 teaspoon a day is expected to last 6 weeks. Each patient will receive supplies for the duration of the study period. While no clear upper tolerable dose in pregnancy has been identified, no increase in the risk of bleeding or complications at delivery has been observed in pregnant women (*n* = 533) receiving 2.7 g/day during the last trimester compared to women receiving olive oil or no supplements (26, 27). An updated review including 70 RCTs (19,927 participants) indicated a reduced risk of preterm birth for those tasking O3FAs compared to placebo or no intervention (28). The dose used in our trial is consistent with a dose that is likely effective and is below the safe maximum dose suggested in these trials.

We will use HMF Maternity Probiotic Formula, a Genestra brand. Each capsule contains 10 billion of four strains of bacteria: *Lactobacillus salivarius* (6.25 billion CFU), *Bifidobacterium animalis* subsp. *lactis* and *Bifidobacterium bifidum* (2.5 billion CFU), and *Lactobacillus paracasei* (1.25 billion CFU). While an updated review of evidence including seven trials (1,647 participants) indicates a possible increased risk of preeclampsia (29), a meta-analysis of several RCTs of probiotic use in pregnancy reported no increased risk to the fetus (30). Furthermore, the probiotic supplement in our trial was specifically labeled for pregnant women, following its use in a double-blind, placebo-controlled clinical trial of 454 pregnant women—no increase in adverse events in comparison to placebo was noted (31).

The placebo capsules will contain 48% maltodextrin, 48% microcrystalline cellulose, 2% magnesium stearate, and 2% silicon dioxide. The capsule shell will be composed of hydroxypropyl ethylcellulose and will be identical in capsule shape and packaging to the probiotics. Participants will be instructed to take one capsule a day with food, 2–3 hours before or after taking an antibiotic in the event that one is prescribed through ancillary usual care received separate from study procedures. Each box of probiotics/placebo is expected to last 30 days. Participants will be provided with enough supplies for the duration of the study period.

#### Concomitant therapies

All concomitant medication, psychotherapy, or natural health products will be allowed during this trial. Participants will be encouraged to continue all other previous treatments at the same dose for the duration of the trial, if possible, but allowed to make changes if recommended by their mental healthcare provider. Participants will be queried at baseline and at each follow-up visit about their use of medication, psychotherapy, or natural health products, and any changes will be recorded, analyzed, and reported.

#### The safety profile of supplements

Marangell et al. studied the impact of O3FA supplementation (2,960 mg/day) on the prevention of postpartum depression. Adverse events reported were mild. Four participants reported a “fishy” aftertaste, one reported mild dyspepsia, and one reported increased stool frequency with loose stools. There were no withdrawals due to adverse events (32). Similarly, Su et al. completed an RCT with 24 participants. No withdrawal of participants due to adverse events was reported. In total, 12/18 participants in the placebo group and 10/18 in the O3FA group did not report any adverse events. Events reported included insomnia (two in the placebo group and three in the O3FA group), nausea (four in the placebo group and six in the O3FA group), and diarrhea (two in the placebo group and one in the O3FA group). The authors noted that most events were mild, with the exception of significant nausea for one participant in the placebo group, which led to the termination of treatment. The authors reported no effects on biological parameters, including abnormal bleeding time or liver function. All newborns were in normal general health at birth (33). Furthermore, Mattes et al. studied O3FA supplementation (3.7 g/day) in 83 pregnant women in the prevention of maternal depression. The authors documented no adverse events (34).

The 2020 meta-analyses by Zhang et al. examined the efficacy and safety of EPA and DHA monotherapy in the treatment of perinatal depression in women with a clinical diagnosis of MDD or symptomatic depression in pregnancy and/or postpartum. Only randomized double/triple-blinded placebo-controlled trials were included in this study (*N* = 8) (35). A total of 638 pregnant women were administered 1–6 g/day a day. The safety of doses of 1–6 g/day was demonstrated in these trials and did not differ significantly between O3FAs and placebo groups within each trial. The most reported side effects were mild and self-limiting. They included nausea, vomiting, increased stool frequency, dizziness, fatigue, and insomnia.

The safety of probiotics has been established. The theoretical risks include infection, adverse metabolic activities, excessive immune stimulation, and gene transfer in susceptible immunocompromised individuals. As far as strains specific to the investigational products, no safety concerns nor adverse effects were observed in clinical trials administering *Lactobacillus salivarius* CECT5713 (36).

No safety concerns have been reported in studies administering *Lactobacillus paracasei* LC01 (37), *Bifidobacterium animalis* subsp. *lactis* (38), and *Bifidobacterium bifidum* (39) to healthy adults.

Long-term safety and efficacy of probiotics administered during the perinatal period were established in a randomized double-blind placebo-controlled study of over 300 pregnant women involving the following strains: *Lactobacillus rhamnosus* GG, *Bifidobacterium lactis* Bb-12, *Lactobacillus paracasei* ST11, and *Bifidobacterium longum* BL999 (40). The authors found no differences in growth or noncommunicable disease prevalence between children receiving perinatal probiotics or placebo.

The safety profile of probiotics is deemed excellent in healthy adults, and pregnancy is not considered a contraindication for their use.

#### Criteria for study withdrawal

All eligible participants will be informed that their participation is completely voluntary, and they can discontinue participation at any time. Participants may withdraw from the study entirely or from specific parts of the study only. If the latter is desired, then follow-up pertaining to other consented components of the study will continue.

Data collected prior to withdrawal may be retained and used in a manner consistent with the study purpose and protocol. The exception is if participants explicitly request the removal of all previously collected information and data, in which case the study team will oblige. Once a participant expresses a desire to withdraw from the study, the research team member in contact with the participant will confirm whether previously collected data can be retained (or should be destroyed) and whether the participant can be contacted in the future for other research opportunities.

The participant will be notified during consent and at the time of withdrawal that any data retained for the analysis cannot be withdrawn after study closure or publication of the study (whichever comes first). Lost to follow-up is defined as enrolled participants that were randomized and completed all parts of visit 1, but completed zero parts of any subsequent visits (2, 4–7). Five attempts will be made to reschedule the study visits for these participants. The number of attempts and modes of communication to reach these participants will be documented. If the missed study visit is not successfully rescheduled within 4 weeks, the visits will be marked as lost to follow-up. Should the participant return to completing the later visits, they will be included in all analyses consistent with the intention to treat. However, if all attempts to reconnect by the study team fail for the missed visit and all remaining visits, and/or if the participant explain that they wish to stop participating in the study, they will be marked as study withdrawal.

All adverse events during the study period will be recorded, and these records will be maintained for 15 years. The clinical trial sponsor will inform the Natural and Nonprescription Health Product Directorate of Health Canada of any serious expected or unexpected adverse events related to the study product immediately if possible and no later than 7 days after becoming aware of the information. Reported symptoms of psychosis (hallucinations, delusions, or disordered thinking) will trigger a referral of participants to the study psychiatrist for assessment and to determine whether they meet the criteria for withdrawal. If a participant reports a significant worsening in symptoms of anxiety or depression, they will be permitted to remain enrolled in the study but will be advised to contact their primary mental healthcare provider for assessment and management. If the mental healthcare provider suggests that psychiatric rescue medication is needed, this clinician will recommend the treatment to the participant. Changes to treatment regimens must be reported to the study team for documentation.

Serious adverse events will also be reported to the research ethics boards that have reviewed the study protocol.

Participants who complete all parts of the study but fail to adhere to regular supplemental intake (defined as missing 7 or more consecutive days), will be included in the study for as long as they wish. Their reasons for not taking the supplements will be documented, and strategies to help overcome this (i.e., forgetfulness) will be made. If the reasons for stopping supplements are allergic reactions or side effects, they will be withdrawn from the intervention, and an adverse report will be made consistent with the safety protocol. If participants still wish to continue with the study, they may do so as part of the control arm.

#### Outcomes measured

As a feasibility study, process evaluation will be our primary outcome. In the first 6 months of the trial, we will monitor the number of patients screened, randomized, and enrolled per month; the average time lapse between screening and enrollment; and the time taken to enroll 15% of our target (i.e., *n* = 15). We will also assess baseline rates of O3FA and probiotics supplementation in this specific cohort prior to randomization. We will identify and document all barriers to meeting the target numbers needed for each study arm and address them appropriately. In patients successfully enrolled, we will also monitor compliance, side effects, and challenges in taking supplements in the intervention groups. We will closely document all issues in adhering to intake instructions and attempt to resolve them on a case-by-case basis (i.e., if forgetfulness is a cause, daily automatic email reminders will be sent to that individual). In Gutopia, attaining a daily fiber target of 35 g/day will be monitored frequently via phone call in the first 6 months or until the patient no longer needs the support. Finally, we will assess retention rates, the proportion of study visits completed, and challenges related to the collection and shipment of stool samples, as well as the completion of questionnaires and dietary recall. All barriers to the timely completion of these tasks will be documented and managed on a case-by-case basis. Strategies to overcome each of these barriers will be proposed in the final write-up of this study.

As part of our secondary aims, we will seek the opportunity to produce preliminary data on the association between maternal microbial response to each intervention and self-reported scores on questionnaires of mental health challenges. This will be for exploratory purposes to identify any signals that will further support the conduct of a larger trial.

##### Mental health outcome

The EPDS and GAD-7 scores will be derived for each subject at enrollment and at every subsequent follow-up. We will calculate the mean and median scores for each group and conduct between-group analyses. Additionally, we will compare the changes between baseline and follow-up scores for each group (within-group analysis), followed by a between-group comparison of these changes.

#### Microbiome profiling

We will be partnering with Zymo Research, a California-based company. Zymo Research is a globally established biotechnology company and industry leader in the fields of epigenetics, microbiomics, and the emerging Next-Gen Sequencing space, with facilities in many countries and a global distribution network. The stool collection kits will be purchased from Cederlane^®^ an Ontario-based distributor of Zymo’s products. The fecal collection kits purchased feature DNA/RNA shield technology, which allows for accurate and safe collection and preservation of samples for downstream analysis. The kits will include a fecal collection tube, a feces catcher, a biohazard bag, gloves, and multilanguage instructions.

The stool will be collected from each patient at baseline and every follow-up visit. The self-collection will occur at participants’ homes, and they will be provided with all shipping materials and instructions to send their collected samples by mail to our institution. All samples will be stored onsite at CCNM according to guidelines provided by Zymo Research. A room in the Schad Naturopathic Clinic that is earmarked for research will be used to store samples; this room is accessible only by keycode, has temperature-monitoring devices, and is marked with applicable biohazard signage. Using their recommended kits, the samples can be stored at ambient temperature for a period of up to 5 years. In total, each patient will contribute six samples throughout the study period. Samples can be shipped as one batch at the end of the study period to Zymo Research in Irvine, California.

This study will not examine blood biomarkers for fatty acid intake.

The following outline analysis is aimed at a larger efficacy trial. Should this feasibility trial succeed, we will continue enrollment with the goal of assessing the efficacy of the interventions and specifically the impact of each on the gut microbiome and mental health outcomes. The following paragraphs provide a brief outline of these secondary aims.

##### Microbial response to diet and supplementation

First, we will assess the microbiome response to each intervention within each group by comparing changes in microbiome composition between baseline (preintervention) and follow-up visits (visits 2–6). The gut profile will be compared between all four arms at each follow-up. The within-group changes from baseline will be compared between groups.

##### Microbiome and mental health

We will measure the interaction between diet and supplementation. The objective is to understand the differences in microbial and metabolic profiles of each group in response to the interventions and the corresponding changes in mental health scores.

##### O3FA and probiotic interaction

The synergism between O3FAs and probiotics has not been defined. We expect that comparing the microbiome between Gutboost and Gutless will yield differences between O3FA alone and O3FA + probiotics on mental health scores.

#### Statistical analysis

Should the trial succeed and we continue to recruit for a larger trial, the following statistical plan will be used to compare mental health outcomes between the groups: we will use mental health scores as the primary outcome and an intent-to-treat approach for these analyses.

Participants lost to follow-up will be assumed to have lost interest/commitment to the study and will be excluded from the analysis. Those who discontinue treatment will still be included.

Between-group differences for EPDS/GAD-7 scores will be analyzed using analysis of covariance (ANCOVA), with group membership as the independent variable, EPDS/GAD-7 as dependent, and pharmacotherapy, exercise status, and any form of psychotherapy as covariates.

The interaction effect between the O3FA and probiotics model will be analyzed and reported in each model. Alternative models will be used if the assumptions of ANCOVA are violated. If differences are found between groups, appropriate *post-hoc* analyses will be conducted to identify where the differences lie. Within-group differences at each time point will be analyzed using repeated measures ANOVA. Data from each follow-up will be compared to baseline scores. EPDS/GAD-7 scores will be the dependent variables, and follow-up times will be our independent variable.

Each stool sample will undergo shotgun metagenomics for taxonomic and functional output readings. A comprehensive analysis of all findings will be conducted by an experienced bioinformatics expert at Zymo Research. All researchers involved in the stool analysis will be blinded to the type of intervention. The relationship between each intervention and the relationship to EPDS and GAD-7 scores will be analyzed and reported by an experienced bioinformatician.

#### Data management

The collection and storage of data will be done according to Good Clinical Practice Guidelines. All study staff involved in the collection or entry of data will be trained by the trial coordinator and will complete ethical conduct of research training (Tri-Council Policy Statement: Ethical Conduct of Research Involving Humans 2). Data from potential and enrolled participants will be stored in the secure platform, Research Electronic Data Capture (REDCap), and a password-protected database on a secure server managed by the Canadian College of Naturopathic Medicine. Data collected at each study visit will be captured using an electronic case report form, which will be entered into the database. Questionnaire completion will be done by the study participants through REDCap. Laboratory data will be entered into the database and double-checked for accuracy by a team member blind to participant allocation. An audit of the data will be completed by a senior member of the research team in 3 months. All information obtained during the trial will be kept in a locked cabinet or on a password-protected computer and destroyed (deleted or shredded) after 15 years.

#### Access to data and dissemination

All personal health information will be kept confidential and only accessed by the research team, unless required by law. This includes an audit by the Canadian College of Naturopathic Medicine Research Ethics Board, the Sunnybrook Health Sciences Center Research Ethics Board, or Health Canada Natural and Nonprescription Health Products Directorate. All data will be maintained on password-protected, secure servers or locked filing cabinets for the required length of time. Following this time, data will be destroyed securely by deleting the digital files or shredding paper documents. No identifying information from any participant will be used in the dissemination of study results. Dissemination of the study findings will occur through publication in open-access journals. Study participants will have the option to be notified of the study findings.

## Discussion

Given the prevalence of PDA and its resulting impact on quality of life and infant well-being, the importance of investigating underutilized and overlooked nonpharmacological options is paramount. Specifically, the need to investigate alternative approaches in comparison to prescription medication and psychotherapy has been identified as an important need by expecting and new mothers. It is anticipated that pregnant women with a history of depression and anxiety will be particularly interested in partaking in this trial, resulting in favorable recruitment rates. Given the positive findings of O3FA and probiotic supplements on mental health symptoms in nonpregnant adults, we expect a similar trend in PDA symptoms, with a low likelihood of adverse events.

While not our main objective, we anticipate a correlation between the microbial composition and probiotics and O3FA supplementation. As several recent studies have demonstrated a positive impact of probiotics (19–21) or O3FAs (22, 23) on mental health outcomes via changes in the gut microbiome, this has yet to be demonstrated in individuals at risk of PDA. Having intervention groups that provide both supplements (Gutboost) versus O3FA alone (Gutless) will allow us to determine potential interaction between these supplements. We anticipate a difference in mental health scores between the Gutboost and Gutless intervention groups. Incorporating a third intervention arm where dietary counseling is provided in addition to supplementation (Gutopia) further allows us to understand the interaction between diet and supplementation. We anticipate differences in mental health scores between Gutopia compared to the other intervention arms. Additionally, having an active control arm can potentially inform the magnitude and directionality of these changes by comparing outcomes in the intervention arms (*N* = 75) to the active control arm (*N* = 25).

Being the first feasibility trial to investigate probiotic supplementation and O3FAs for PDA symptoms, this study lays the foundation for larger, more powerful studies to further contribute evidence for the efficacy of this potential preventative treatment option. This type of evidence creates a rationale for the inclusion of nutrition professionals in mental healthcare teams and the use of dietary counseling in the treatment of PDA. Nutrition interventions can be low-risk, acceptable to patients, cost-effective, and may have additional benefits to overall health, which is particularly important for our target population.

## Author contributions

FG: Writing – original draft, Writing – review & editing. KC: Funding acquisition, Methodology, Supervision, Writing – review & editing. SG: Methodology, Supervision, Writing – review & editing. NE: Conceptualization, Funding acquisition, Methodology, Project administration, Supervision, Writing – review & editing.
