# Supplementary material for: Targeting maternal gut microbiome to improve mental health outcomes—a pilot feasibility trial
Source: Front Psychiatry. 2024 Aug 7;15:1414291. doi: 10.3389/fpsyt.2024.1414291 (PMC11335611; doi:10.3389/fpsyt.2024.1414291)
Supplement: Supplementary file 1 [file DataSheet_1.pdf]

Questionnaire	Version Used	Reliability	Validity
GAD-7	Spitzer RL, Kroenke K, Williams JB, Löwe B. A brief measure for assessing generalized anxiety disorder: the GAD-7. Arch Intern Med. 2006;166:1092-7.	Research indicates the GAD-7 shows excellent internal consistency (Spitzer, Kroenke, Williams, & Löwe, 2006)<br><br>The GAD-7 shows good test-retest reliability (Spitzer et al., 2006)	The GAD-7 is a useful tool with strong criterion validity for identifying possible cases of GAD (Spitzer et al., 2006)<br><br>Löwe et al. (2008) substantiated the 1-dimensional structure of the GAD-7 and its factorial invariance for gender and age.<br><br>The GAD-7 yielded significant intercorrelations with the PHQ-2 and the Rosenberg Self-Esteem Scale (Löwe et al., 2008)<br><br>Higher GAD-7 scores correlate with disability and functional impairment (in measures such as work productivity and health care utilization) (Spitzer et al., 2006; Ruiz et al., 2011)
EPDS	Cox, J.L., Holden, J.M., and Sagovsky, R. 1987. Detection of postnatal depression: Development of the 10-item Edinburgh Postnatal Depression Scale. British Journal of Psychiatry 150:782-786. K. L. Wisner, B. L. Parry, C. M. Piontek, Postpartum Depression N Engl J Med vol. 347, No 3, July 18, 2002, 194-199.	The EDS showed high test–retest reliability and high concurrent validity with the SCL-90 anxiety and somatization subscales (Bergink et al., 2011).	Research has shown that administering the EPDS for fathers is valid, and that a score greater than 10 has a sensitivity of 89.5% and specificity of 78.2% (Bergink et al., 2011).<br><br>The EPDS administered in the 1st week postpartum was predictive of maternal mood at 4 and 8 weeks postpartum (Dennis, 2004).
SISQS	Snyder E, Cai B, DeMuro C, Morrison MF, Ball W. A new single-item sleep quality scale: results of psychometric evaluation in patients with chronic primary insomnia and depression. *J Clin Sleep Med.*2018;14(11):1849–1857.	Test-retest reliability (intraclass correlation coefficient) was .62 during a 4-week period of sleep stability in patients with insomnia and .74 in stable patients with depression (1 week) (Synder et al., 2018).	Concurrent criterion validity (correlation with measures of a similar construct) was demonstrated by strong (inverse) correlations between the SQS and MQI (week 1 Pearson correlation −.76) and PSQI (week 8 Goodman-Kruskal correlation −.92) sleep quality items in populations with insomnia and depression, respectively (Synder et al., 2018).
PPAQ	Chasan-Taber, L., Schmidt., M.D., Roberts, D.E., Hosmer, D.., Markenson, G., Freedson, P.S. (2004). Development and	For test-retest reliability, r values were respectively 0.961, 0.934, 0.957 and 0.981 for self-reported sedentary, light,	For validity, the Pearson's correlation coefficients between the Pregnancy Physical Activity Questionnaire and long form of the International Physical Activity Questionnaire ranged from

	Validation of a Pregnancy Physical Activity Questionnaire. Medicine & Science in sports & Exercise, 26(10), 1750-1760.	moderate, and vigorous activity, respectively. Intraclass correlation coefficient scores ranged from 0.924 to 0.993 (Cirak et al., 2015).<br><br>The ICC values of Reliability were: total score (0.77); sedentary activities (0.87); light-intensity activities (0.76); moderate-intensity activities (0.76); vigorous-intensity activities (0.70) (Santos et al., 2023).<br><br>The correlation coefficient for total physical activity was r = 0.96. The reproducibility of the results obtained from PPAQ questionnaire was the highest for inactivity and moderate activity (r = 0.96). The analysis according to the type of activity showed that the reproducibility was the highest for occupational activity (r = 0.98) (Suliga et al., 2017)	moderate (r = 0.329) to high (r = 0.672) (Cirak et al., 2015).<br><br>For criterion validity was obtained a coefficient correlation of r = -0.030, considered weak and negative, for total activity (Santos et al., 2023).
CFPB-FWB	CFPB Financial Well-Being Scale, Scale Development Technical Report https://sjdm.org/dmidi/files/CFPB_Financial_Well-Being_Scale_Technical_Report.pdf		
